# MEMS-Based Power Generation Techniques for Implantable Biosensing Applications

**DOI:** 10.3390/s110201433

**Published:** 2011-01-26

**Authors:** Jonathan Lueke, Walied A. Moussa

**Affiliations:** Department of Mechanical Engineering, University of Alberta, University of Alberta, Edmonton, Alberta T6G 2G8, Canada; E-Mail: lueke@ualberta.ca

**Keywords:** power micro-generation, implantable biosensors, photovoltaic, thermovoltaic, micro fuel cell, electrostatic, electromagnetic, piezoelectric

## Abstract

Implantable biosensing is attractive for both medical monitoring and diagnostic applications. It is possible to monitor phenomena such as physical loads on joints or implants, vital signs, or osseointegration *in vivo* and in real time. Microelectromechanical (MEMS)-based generation techniques can allow for the autonomous operation of implantable biosensors by generating electrical power to replace or supplement existing battery-based power systems. By supplementing existing battery-based power systems for implantable biosensors, the operational lifetime of the sensor is increased. In addition, the potential for a greater amount of available power allows additional components to be added to the biosensing module, such as computational and wireless and components, improving functionality and performance of the biosensor. Photovoltaic, thermovoltaic, micro fuel cell, electrostatic, electromagnetic, and piezoelectric based generation schemes are evaluated in this paper for applicability for implantable biosensing. MEMS-based generation techniques that harvest ambient energy, such as vibration, are much better suited for implantable biosensing applications than fuel-based approaches, producing up to milliwatts of electrical power. High power density MEMS-based approaches, such as piezoelectric and electromagnetic schemes, allow for supplemental and replacement power schemes for biosensing applications to improve device capabilities and performance. In addition, this may allow for the biosensor to be further miniaturized, reducing the need for relatively large batteries with respect to device size. This would cause the implanted biosensor to be less invasive, increasing the quality of care received by the patient.

## 1. Introduction

Microelectromechanical Systems (MEMS)-based sensors are gaining notoriety for biosensing applications due to their small size, low power consumption, and high integratability into microelectronic systems for implantable sensing applications. Generally MEMS devices have one, if not all, of their major dimensions in the micrometer range, many being not much bigger than a few tens of cubic millimetres when packaged. For implantable applications, MEMS-based devices can be used in a multitude of roles such as sensing a variety of different phenomena including physical loads on joints or implants, vital signs, measuring bone density, or osseointegration; enabling targeted drug delivery; and diagnosis through lab-on-a-chip devices. This is attractive for bio-applications since these MEMS-based devices are less invasive to implant than larger macro-scale sensing devices, allowing them to be implanted at a variety of locations in the body where macro-scale devices may not be suitable. In addition, MEMS-based devices have very low power consumption, which when coupled with active power management, allows the implantable MEMS-based biodevices to operate for long periods of time.

The most common method to power MEMS-based *in-vivo* devices is a conventional or thin film battery. Normally, the battery system becomes a limiting factor to the lifespan and applicability of many microbiosensors. Although some biocompatible batteries may have long life spans, the battery will eventually require replacement or recharging. For short term applications, a battery may thus provide a sufficient device lifespan, but for long term or high duty cycle applications, alternative power schemes may be preferable to replacing dead batteries, especially if the replacement/recharge procedure is invasive. For example, pacemakers are a common implantable system that requires an independent power source that functions completely autonomously from the outside world. The current standard for pacemaker operation is to utilize a high-life battery that supplies approximately 0.65 to 2.8 Ampere hours for 5.1 to 9 years [1]. Eventually, the battery for this system will need to be replaced, requiring additional surgery. Although a pacemaker is not necessarily a biosensing device, or a MEMS scale device, the power supply has been augmented by an electromagnetic-based MEMS generator. Roberts *et al.* [2] developed a system by which an electromagnetic MEMS-based generator captures the vibrational energy produced by the heart muscle to generate power to supplement the pacemaker’s internal battery. In initial clinical trials, it was possible to produce up to 17% of the energy required to operate a conventional pacemaker [2]. Further development of this technology may be able to eliminate the costly and invasive surgeries required to maintain the pacemaker, both decreasing medical cost and improving the quality of care for the patient. A direct analogy can be drawn to the possible applications of this strategy to implantable biosensing. Any number of implantable biosensing platforms could have their power systems replaced or augmented by MEMS-based power generators. The addition of MEMS-based generators to the conventional power systems of these sensors would allow for increased lifespan and the ability to add components to the sensing platform that may have been too energy-costly to initially add to the system. Additional hardware could also be integrated into these sensing packages, allowing for wireless communications and on-board computing to further increase the functionality and usefulness of said MEMS-based implantable sensors.

Although micro scale power generation has many forms, the same general operational principles are used as in macro scale power generation—a specific form of energy is converted into electricity via a specific physical phenomenon. The major difference between micro and macro scale power generation is the scale at which the generation takes place. As you decrease the size of a device into the micro regime; the relative strengths of all physical forces changes. For example, highly length-dependant forces, such as electrostatic forces, become increasingly dominant over gravity. Therefore, the MEMS devices are more likely to be influenced by what would be considered to be ambient forces on a macro scale. Ambient forces and energy are non-negligible for MEMS devices, and in some cases, this ambient energy can be harvested by micro generation techniques to produce electricity. Ambient light energy may be converted into electricity using photovoltaic cells [3–13]. To convert ambient thermal energy to electricity, thermoelectric generators may be used [14–22]. In addition to scavenging techniques, chemistry-based techniques, such as micro fuel cells [13,23–35] can be used to supplement battery-based power schemes. Micro fuel cells use a variety of electrochemical reactions to produce electricity. Some micro fuel cells can regenerate their fuel and oxidation agents through the electrochemical reactions that take place within the fuel cell allowing for long term operation [13,23–35]. Vibration is converted to electricity via electrostatic [4,36–47], electromagnetic [2,48–54], and piezoelectric microgenerators [55–67].

In the following sections each of the above micro-generation methods will be examined in detail. The relative applicability of these methods will be evaluated and discussed, highlighting both strengths and weaknesses for various generation physics in various applications. It will be shown that for various ambient energy types, quantities of ambient energy, and environmental conditions certain methods of MEMS-based generation will be more suitable for generation of power for implantable biosensing applications.

## 2. Methods of Micro-Generation

### 2.1. Photovoltaic Generation

Photovoltaic cells are the most recognizable energy scavenging technique currently in use, both in small and large scale applications, ranging from hand-held calculators to commercially generated electricity. MEMS-based solar cells are based upon electronic asymmetry, such as a p-n junction found in semiconductors. As this electrical asymmetry is illuminated, incident photons cause electron hole pairs to form, promoting local electron mobility. If connected to a load, free electrons will flow through the load and then back to the solar cell, where vacant electron holes are located [3]. In order for photovoltaic cells to be efficient, they must be placed in direct, bright sunlight. Without direct, high intensity light, the generating capacity of a photovoltaic cell can diminish significantly from 15 mW/cm^2^ in direct sunlight to 10 μW/cm^2^ in normal office lighting [4]. Photovoltaic cell materials need to be carefully chosen, since the measured output power can vary over three orders of magnitude at low illumination levels [5]. MEMS-based solar cells can be fabricated from a variety of materials, including single crystal silicon, thin film polysilicon, gallium arsenide, cadmium telluride, hydrogenated amorphous silicon and ferroelectric films such as lead lanthanum zirconate titanate (PLZT) [3,6,7]. These materials are chosen due to their suitable semiconductor band gaps of 1.4–1.6 eV [3]. Solar cells using the hydrogenated amorphous silicon, such as those developed by Lee *et al.* [3], produce a usable amount of electrical power, due to the large band gap (1.55 eV) present in the hydrogenated amorphous silicon. The solar cell can produce open circuit voltages of 1.5 V per cell in series and short circuit current of 0.28 μA per cell.

Indium gallium arsenide photogenerators have been developed for use in fiber optic networks to power optical switches and controllers far down-cable. The ability of photoelectric generators based upon harvesting light energy from fiber optic cables may allow for the use of photoelectric-based generators *in vivo*. The required high intensity light may be channeled to the subcutaneous implant by collector and fiber optic cable, possibly removing the requirement for direct light. High efficiency photodiodes are available for this application [8–10] which convert the long wavelength light (1,300–1,550 nm) into electricity. These photodiodes are high efficiency, but the voltage available from these relatively small band gap diodes is too small for many switching and controlling applications. Dentai *et al.* connected 30 diode segments in a favorable configuration in order to increase the overall electricity generation from approximately one volt, to 10.5 V at 500 μW, converting 1,554 nm incident light [11]. The photodiodes are arranged in pie-segments, 30 pie-shaped photodiodes that are arranged in a complete circle. This arrangement allows for increased conversion area, reduction of contact resistance, the ability to use anti-reflection coatings on the incident surface of the photodiode, and the ability to metalize the backside of the photodiode to allow unconverted light to have a second pass through the photodiode [11]. Similar photovoltaic cells fabricated from gallium arsenide have generated upwards of 1 W of electrical power using concentrated incident light as a power source [12].

Solar-based schemes also can use photosynthesis as the driving force behind micro-generation in a hybrid photoelectric fuel cell [13]. A photosynthetic electrochemical fuel cell has been developed, where sub-cellular thylakoid photosystems isolated from spinach cells provide the chemical reactions necessary to generate electricity. During photosynthesis, water is split to produce protons (H^+^) and electrons which are both collected by the anode of the cell. The current that is drawn from the anode is then used in a device, and then returned to the cell through the cathode of the cell, either reducing O_2_ or regenerating the ferricyanide used in the cell as charge carriers. This process not only produces electricity for use in a device, but regenerates the chemical reagents used in the initial reaction. This photo-driven fuel cell can produce power densities of up to 5.4 pW/cm^2^ [13]. For biological applications the photosynthesis-based micro fuel cell is attractive for its biocompatibility, having no bio-incompatible fuels or chemical reactions.

### 2.2. Thermoelectric Generation

Direct thermoelectric generators utilize the Seebeck Effect to generate electricity. The Seebeck Effect is the direct conversion of a temperature difference into an electrical potential between a material pair junction [14,15]. Thermoelectric generators made from thermocouples made of aluminum and n-poly-Si, p-Bi_0.5_Sb_1.5_T_3_, and n-Bi_0.87_Sb_0.13_ were developed by Huegsen, Woias, and Kockmann [14,15]. In this case, thin film thermocouples of the above composition were fabricated, and then connected in series to form thermopiles. In order to maximize the power generation of the thermoelectric generator, a large thermal contact area is required. To allow for a large thermal contact area, the heat flow path is guided by thermal connectors to be perpendicular to the surface of the thermopiles. This method shapes the thermal profile of the thermopile, allowing for 95% of the entire temperature difference to be located between the two thermopile junctions, which in turn maximizes the possible heat that can be used in conversion. Power factors as high as 3.63 × 10^−^^3^ W/mm^2^K^2^ and 8.14 × 10^−^^3^ W/mm^2^K^2^ can be achieved through this method [14,15]. Direct thermoelectric generation is considered to be an energy scavenging technique, since waste heat is an abundant energy source. As long as heat energy is available to the microgenerator, energy conversion will continue without interruption. The maximum energy that could be converted from thermal to electric energy is determined by the Carnot efficiency of the generating situation [16]. Since the efficiency of the thermal-to-electricity conversion is limited by the Carnot efficiency, small thermal gradients will not be efficient in producing electricity. For thermopile arrays, it has been reported for temperature differences of 180 °C (200–20 °C) the efficiency of the thermopile array is 10%. In comparison for a temperature difference of 20 °C (40–20 °C), the same thermopile array has an efficiency of 1% [16,17].

Thermoelectric generators using the human body as a heat source have been explored by Leonov *et al.* [18]. With a wide range of tissues and fluids with each having their own unique material and thermal properties, it was found that the human body has an inherent non-uniform temperature distribution. Thermal profiles in different regions of the body may vary due to proximity to blood vessels and function of surrounding tissues and organs. The variation of thermal characteristics of the body extends even to the skin and extremities of the body. Areas such as wrists and ankles will be considerably warmer due to the proximity of major blood vessels to the skin and the external environment, therefore it is advantageous to strategically place thermoelectric generators in these locations to maximize generation [18]. The microgenerator itself is a microfabricated array of polysilicon-germanium (poly-SiGe) thermocouples, which are sandwiched between two silicon wafers and interconnected in series to form thermopiles. The microgenerator was integrated into a wrist-mounted package approximately 3 × 3 × 1 cm^3^ in size, in order to allow for heat absorption directly from the radial artery of the wrist [18]. These poly-SiGe thermopiles can produce upwards of 4.5 μW/cm^2^ of power on the radial artery [18]. This location was chosen since it has the maximum temperature difference between the body and the outside environment, thus maximizing generation. A second thermoelectric generator was produced with commercially available BiTe thermopiles that were developed for the same application for comparison, using a similar wrist mounted package. Poly-SiGe thermopiles are a more cost effective and mature technology in comparison to the BiTe thermopiles. However, the BiTe thermocouple-based thermoelectric generator was able to produce on average 100 μW of power, which was then stored in 2 NiMH batteries. The BiTe microgenerator was composed of 128 BiTe thermocouples, forming 48 thermopiles, taking a volume of 8.2 × 8.9 × 2.4 mm^3^ [18]. In comparison, the BiTe-based thermoelectric generator produced a power density of approximately 571 μW/cm^2^, in comparison to the poly-SiGe thermoelectric generator that produced 4.5 μW/cm^2^. The volume savings of using a more effective thermocouple is significant in this case, the BiTe-based thermogenerator producing a much higher power density with a much smaller device. This technology has been adapted for wrist watches, and is currently being used by multiple commercial watch companies in thermo-electrically driven watches. It has been reported that up to ten similar thermoelectric modules as the above are used to produce the required electricity to power these watches [16].

In addition to direct thermoelectric conversion, there has been work dedicated to the direct conversion of heat to mechanical actuation, which is then converted into electricity using a secondary conversion mechanism [19]. Instead of converting heat energy to electricity and then using the electricity to actuate a MEMS device, careful selection of geometries and materials can allow for controllable actuation directly from thermal expansion. This can allow for both in plane and out of plane linear actuation. To achieve out of plane displacement, two similarly shaped cantilever beams are fabricated one on top of the other. The top beam is approximately 25% thinner than the bottom beam. When connected to each other at their respective free ends forming a U-shape, the thermal expansion of these cantilever beams becomes linked. Therefore, as this structure is heated, asymmetric thermal expansion between the two connected beams allows for the U-shaped structure to actuate out of plane. To achieve in plane actuation, cantilever beams are connected perpendicularly to an actuator. The beams are constrained *versus* axial thermal expansion. When heated, these beams will expand, and eventually will start to buckle. This symmetric thermal expansion and buckling will actuate the central beam in-plane [19]. In addition, rotation may be achieved through the use of micro heat engines, including Brayton Cycle micro-gas turbine engines [20] and Otto Cycle based heat engines [21], both directly converting hydrocarbon fuels into rotary motion. Although these thermal-based actuation schemes do not directly produce electricity, it is possible to use these systems as another actuation method for various other generation techniques. For example, the linear actuation schemes can be used to actuate electrostatic, electromagnetic, and piezoelectric based generation schemes. The rotary actuation schemes can be used to actuate rotary electromagnetic MEMS-based generation schemes.

Thermoelectric generation also entails the use of heat engines to produce electricity on a micro scale. Heat engines, such as the P^3^ micro heat engine developed by Whalen *et al.* [22], can convert hydrocarbon fuels to electricity on a micro scale. The P^3^ heat engine is comprised of two major systems: A combustion chamber that produces heat for the engine and a two-phase working fluid that provides a pressure load to a piezoelectric membrane when heated. The heat supplied to the two-phase working fluid causes the fluid to expand and apply pressure to a piezoelectric membrane. The piezoelectric membrane converts that mechanical strain into electricity via the piezoelectric effect. The heat engine has a four phase working cycle: Compression, isothermal high temperature heat addition, expansion, and isothermal low temperature heat rejection. The piezoelectric membrane is deflected during the compression and expansion phases experienced by the two-phase working fluid. For characterization, a resistance heater was used to provide the thermal energy required in order to actuate the piezoelectric membrane. The resistance heater was operated using a square wave, with a 1 ms pulse width, at voltage amplitude of 3.2 V. The resistance heater, having a resistance of 1.7 Ω, delivered 1.45 W of thermal energy to the working fluid. The piezoelectric membrane produced a voltage varying between 63 and 135 mV at a frequency of 240 Hz. With a load resistance of 14 kΩ, the P^3^ heat engine produced 0.8 μW at these conditions.

### 2.3. Micro-Fuel Cells

Micro fuel cells operate by harvesting electrons from controlled electrochemical reactions. Depending upon the fuel and oxidizing agents reacting in the micro fuel cell, it can be considered either a regenerative or non-regenerative generation technique. If the electrochemical reactions that take place are self-sustaining, such that the reactants are not irreversibly consumed, the fuel cell is regenerative. For example, glucose-based, self contained fuel cells [23] are completely regenerative, able to operate for extended periods of time without outside intervention. The electrochemical reactions that take place in the glucose-based fuel cell can occur continuously without exhausting fuel or oxidation chemical supplies. Non-regenerative fuel cells usually have solid oxide fuels, methanol, or hydrogen as a fuel utilizing a non-reversible reaction to produce free electrons. These fuels have higher energy densities but consume the fuel as the electrochemical reaction takes place. These non-regenerative fuel cells produce power as long as there is fuel present.

Glucose based micro fuel cells for biomedical applications are well researched. This type of fuel cell relies on the electrochemical reaction of oxygen and glucose – two substances commonly found in the body. For *in vivo* applications, a glucose-based fuel cell could potentially have an unlimited fuel supply [23]. Glucose-based fuel cells can be categorized into three specific types: enzymatic, microbial and abiotic. Enzymatic refers to glucose fuel cells that employ enzymes in order to facilitate the required chemical reactions to produce electricity. Microbial glucose fuel cells employ specific micro-organisms that convert the glucose found in a system to electricity [13]. Abiotic fuel cells use non-biological catalysts in order to ensure that conversion of glucose to electricity takes place. As in all fuel cells, electricity is generated by the electrochemical reaction of a fuel and an oxidant at two separated electrodes. Regardless of the fuel or the method of which is taken to catalyze the reaction, electrons released from the oxidation of the fuel are collected by the anode, flow through the load to the cathode, upon where a terminal electron acceptor is reduced. The electron flow is driven by the difference in electrochemical potential of the anode and cathode redox pairs [24]. One molecule of glucose can be completely oxidized into carbon dioxide and water, releasing 24 electrons per molecule, as shown below [24]:

(1) $$\mathrm{Annode}:C_{6}H_{12}O_{6}+24OH^{-}\to 6CO_{2}+18H_{2}O+24e^{-}$$

(2) $$\mathrm{Cathode}:6O_{2}+12H_{2}O+24e^{-}\to 24OH^{-}$$

(3) $$\mathrm{Overall}:C_{6}H_{12}O_{6}+6O_{2}\to 6CO_{2}+18H_{2}O$$

Theoretically, it is possible to collect and use all 24 electrons that are generated in this reaction. However, in practice this has not been achieved [24]. In addition, this single reaction would generate a theoretical voltage of 1.24 V [24]. The major attraction for the glucose fuel-cell is the fact that the fuel and the reaction products are highly biocompatible; therefore it can be considered for *in vivo* MEMS-based applications. Glucose-based micro fuel cells have been reported to produce 50 μW/cm^2^ to 430 μW/cm^2^ for long-term constant generation [25].

Solid oxide fuel cells (SOFC) have also been developed [26]. These fuel cells use a novel microfabrication method of directly printing the anodes and cathodes used in this system in specific configurations. They are deposited using a direct-write system, where suspensions of 55 wt% NiO/45 wt% YSZ (anode) and (La_0.8_Sr_0.2_)_0.98_MnO_3_ (cathode) powders are deposited as a paste onto the fuel cell’s surface through a robotically controlled micronozzle system. This allows for a variety of possible electrode configurations, maximizing electrode/reactant surface area contact. In addition, by using these specific electrode materials, it is possible to use hydrocarbons as a fuel in this fuel cell since the operational temperature of this fuel cell can be significantly higher than fuel cells composed of other materials. Hydrocarbon-based fuel cells have higher energy densities than other fuel cells [27]. A mixture of methanol and air is used as the fuel in this fuel cell which produces an open circuit voltage of 0.9 V and a peak power density of 2.3 mW/cm^2^ at 700 °C. This type of micro fuel cell has a much higher operational temperature than the previous ones, but produces much more usable energy [26]. When applied to other types of fuel cells, this approach may help with further miniaturization and optimization of power output, especially for size critical *in-vivo* applications.

Direct Methanol Fuel Cells (μDMFC) are another type of fuel cell of interest. Common power generation values range from 200 mV–1 V, and microwatts of power. For example, a micro fuel cell developed by Sim, Kim and Yang for the biological application uses methanol as a fuel [28]. Although methanol is toxic to biological systems, it is a good example of the technology. The operation of these systems is based on the electrochemical reaction shown below:

(4) $$CH_{3}OH+H_{2}O\to CO_{2}+6H^{+}+6e^{-}$$

The μDMFC is a type of Proton Exchange Membrane Fuel Cell (PEMFC) [29], which not only relies on the collection of electrons at the anode to produce a current, but the migration of protons (H^+^) to the cathode where catalysts allow the hydrogen to form water, completing the electrical circuit. A limiting factor to the application of these devices in their current state is their size. Currently, a common size for these devices is 16 × 16 × 1.2 mm, which may be too large for some implantable biosensing applications. Another limiting factor to the lifespan of the device, barring any physical damage, is the amount of fuel available to the system [30]. As long as the fuel cell has a sufficient supply of fuel, it will produce electricity uninterrupted. Generally, this type of fuel cell is constructed of two silicon wafers with a membrane electrode assembly patterned onto a membrane, such as Nafion, sandwiched in between. The silicon wafers are micromachined with through-holes in order to allow both the methanol fuel and oxygen catalyst to reach the membrane/electrode assembly to allow for the electrochemical reaction to take place [29]. The through hole or microchannels are designed to be very small, roughly 80 × 80 μm, to ensure that the capillary forces allowing the methanol to be passively transported to the membrane/electrode assembly are prevalent over gravity forces, which would otherwise prevent fuel flow in certain orientations of the micro fuel cell [31]. This type of passive μDMFC is able to produce 9 mW/cm^2^ for about 50 minutes operation—the time required to exhaust the methanol fuel source [29].

Motokawa *et al.* [32] have developed a novel parallel microchannel system for μDMFCs that allows for a greater active area on the membrane interface that transports protons. The micro fuel cell is composed of two parallel microchannels, connected on the top surface by a DuPont Nafion 112 proton membrane. The multiple anodes and cathodes used in this micro fuel cell are located on the bottom and sides of the microfluidic channels, which allow for high efficiency collection of electrons by the anodes and high efficiency transportation of protons to the cathodes. The travel distance from anode to cathode is very short; this causes the system to be less sensitive to ohmic impedance [32]. In addition, this approach isolates the fuel and oxidant in the fuel cell, preventing any cross-mixing of fuel and oxidant that may occur in other fuel cells. This novel technique prevents some traditional problems in micro fuel cell design, but does not produce as much power as other designs, only producing 0.78 mW/cm^2^ [32]. In addition to travel distance, the geometry of the fuel cell plays an important role in the efficiency and generation potential of the μDMFC. Generally, the anode flow field plate is designed to maximize the surface area upon which the required electrochemical reactions take place. By maximizing the surface area upon which the reaction can take place, using a double serpentine structure rather than a pin-type flow plate, the peak power output of the μDMFC can be increased by upwards of 20.7% [33].

In addition to previous schemes used to increase the surface reactive area of fuel cells, stacks of fuel cells can be arranged in a “flip flop” configuration [34] where a common bipolar plate, containing both an anode and cathode, can be used to achieve long continuous stacks of fuel cells. By connecting one anode side of a common plate to a cathode side of a different common plate, long stacks can be created, increasing the generation potential of that single, “flip flopped”, fuel cell. This scheme minimizes the connection resistance of the system. This scheme, when fueled with 2 M methanol, produced 2.7 V of open circuit voltage, with a peak power output density of 2.2 mW/cm^2^ [34].

Carbon Nanotubes have been perused as both a catalyst support layer and a gas transport method for micro-fuel cells [35]. A honeycomb-type arrangement of carbon nanotubes is used to transport both the fuel and oxidant between reaction sides of the fuel cell. In this case, an air/hydrogen mixture is used as fuel. Studies conducted by Kuriyama *et al.* [35] focused on demonstrating that carbon nanotubes were a viable structural material for both material transport and as a support layer. The micro fuel cell using carbon nanotubes as a transport medium for catalysts was able to produce an energy density of 0.75 W/cm^2^ [35]. The carbon nanotube transport system also allowed for a more uniform and predictable transportation of materials around the fuel cell. In traditional fuel cells, pressure driven diffusion across a membrane is the primary method of reactant transportation. With carbon nanotubes, it is possible to easily transport materials without a pressurizing mechanism, allowing for greater reliability and standardization of specifications between similar fuel cells [35].

### 2.4. Electrostatic Vibration-to-Electricity Conversion

**Figure 1.**
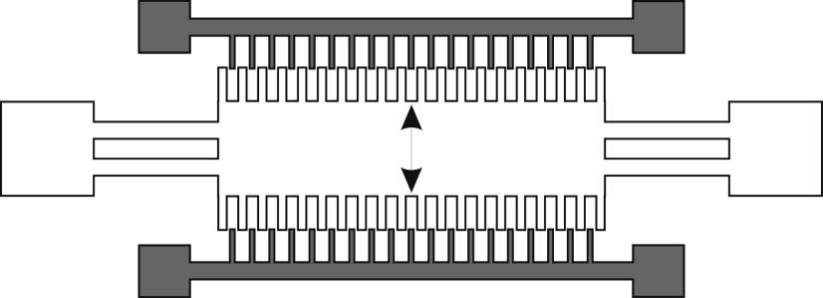
Schematic of an In-Plane Overlap Electrostatic Micro Generator, Direction of Travel Indicated [42].

Electrostatic vibration-to-electricity energy harvesting most often utilizes a comb drive to generate electricity from a base vibration. With these devices, power is generated through a vibration-driven capacitance variance which causes charge transfer and current flow. The capacitors must be held at a constant charge to promote power generation, therefore a polarization source must be present in order to generate additional power. The charge required for the system to operate can be supplied actively from a power source or passively through use of an electret layer [36,37] or a charge pump [38,39]. With an electret-driven microgenerator, an electret layer provides the necessary polarization of the variable capacitor. The electrets are microfabricated from silicon wafers, with deposited layers of silicon oxide and silicon nitride. The wafer is subject to a corona charge, which deposits a significant amount of charge in the silicon nitride layer. After a heat treatment, the charge is trapped within the electret. The average lifetime of the electret under regular operation is approximately 50 years [40]. The charge quantity from an electret directly influences the power generated, up to as much as a few orders of magnitude. A 10 V electret will allow a electrostatic generator to produce 2 nW continually, while a 100 V electret will allow a electrostatic generator to produce upwards of 5 μW [37]. The charge pump is functionally different than an electret, but performs the same task. Instead of having a large amount of charge stored and slowly released over time to polarize the variable capacitors, a charge pump, once primed with an externally supplied charge, will siphon the required energy from the energy generated to maintain the generation cycle. To work effectively, the charge pump requires a flyback circuit and a charge reservoir, such as a battery or capacitor, to prevent charge saturation [39]. Once operating, the charge pump will continually charge the variable capacitors until either a lack of vibration or some other interruption occurs disrupting the cycle long enough for a complete draining of the charge reservoir [39]. Generally, the concept of generating power through electrostatic generation can be summarized in three steps: charge the variable capacitor when the capacitance is high, reduce the capacitance of the variable capacitor through mechanical vibrations, and discharge the capacitor when it is suitable to do so [41]. There are three different types of electrostatic generators which differ by actuation direction, as shown below. The generator shown in Figure 1 is referred to as an in-plane gap closing electrostatic generator. This generator develops a capacitance variance by vibrating in the plane of the device in the direction shown in the Figure 1.

**Figure 2.**
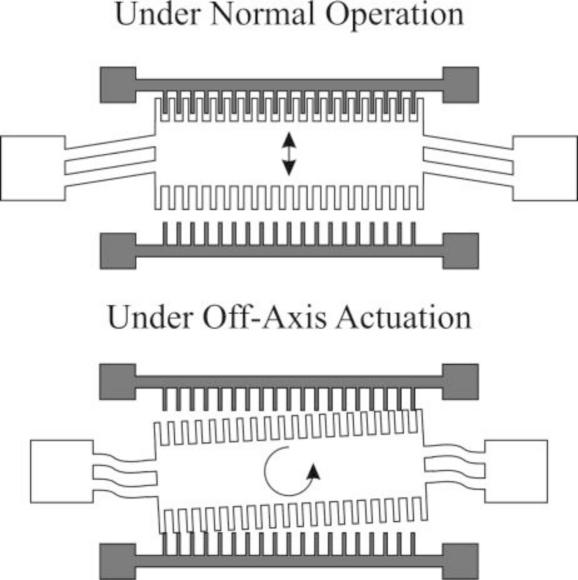
Detrimental Rotation of the In-Plane Overlap Electrostatic Micro Generator [42].

This motion causes the overlap area of the teeth of the comb drive to vary, thereby causing the required capacitance change. The actuation of this device is limited by the spacial gap in the direction of the actuation. In order to prevent damage to the structure, either through impact or stiction, mechanical stops must be fabricated [4]. The mechanical stops limit the minimum dielectric gap in the interdigitated fingers, thereby determining the maximum capacitance of the system. As for power generation, this device can produce up to 20 μW/cm^2^ [4]. The potential generation for this type of electrostatic microgenerator has been shown through simulation to be upwards of 10 μW of power, driven at 120 Hz, under a 3.5 m/s^2^ acceleration [43]. However, due to the design of the comb drives involved off-axis actuation can cause rotation, which promotes electrical contact, shorting, and stiction, as shown in Figure 2.

**Figure 3.**
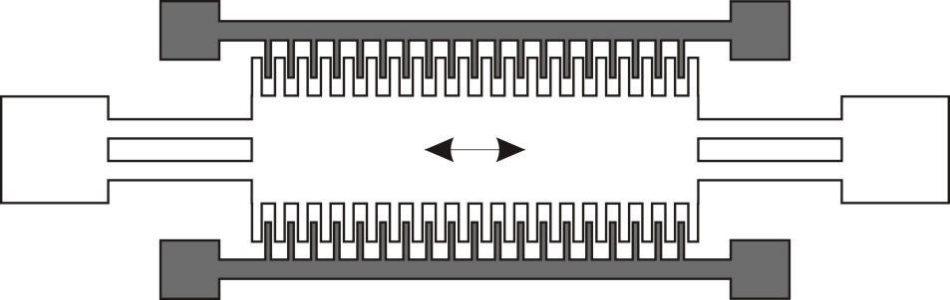
Schematic of an In-Plane Gap Closing Electrostatic Micro Generator, Direction of Travel Indicated [42].

The in-plane gap closing electrostatic microgenerator, as shown in Figure 3, is of the same configuration as in-plane overlap electrostatic microgenerator; however the actuation direction is perpendicular within the same plane. With this device, the capacitance variation is driven through varying the gap between the teeth of the combs.

As before, this device has the same minimum gap restriction, requiring mechanical stops to prevent damage to the system. It is reported by Roundy *et al.* [4] that this design is more manageable and less prone to detrimental in-plane rotation, and therefore, was chosen to be optimized. The in-plane gap converter, once optimized, was able to generate up to 116 μW/cm^2^ vibrating at 2.25 m/s^2^ at 120 Hz [4]. Murillo *et al.* [44] have developed an in-plane closing gap electrostatic microgenerator that can produce 76.67 nW of power at a frequency of 100 Hz. The strength of this electrostatic microgenerator system is the array-like integration which was the focus of Murrilo *et al.’s* research. One hundred microgenerators were integrated into a chip an area of 2.84 × 3.67 mm, increasing the power generation from 76.67 nW to 0.958 μW [44].

**Figure 4.**
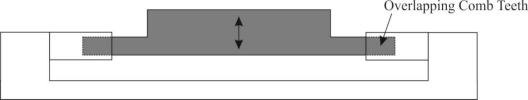
Schematic of an Out-Of-Plane Gap Closing Electrostatic Microgenerator, Direction of Travel Indicated [42].

The last design, as shown in Figure 4, is the out-of-plane gap electrostatic generator. It is of similar form to the previous in-plane electrostatic generators, but is actuated out of plane. As in other iterations of this generation scheme, the out of plane actuation provides the nessciary capacitance change to produce electricity.

However, the out-of-plane gap generator is greatly influenced by thin-film damping and stiction. For this device to produce appreciable power, it must be packaged in a vacuum to avoid thin film damping, in which case the power generation will improve from 1 nW/cm^2^ to 20 μW/cm^2^. Depending on the application, packaging the generator in a vacuum may or may not be possible. In addition, to further make this device viable, mechanical stops would have to be fabricated in order to prevent the out-of-plane gap converter from contacting the substrate, thereby shorting and causing stiction. These mechanical stops are extremely difficult to fabricate since there is no geometrical freedom to produce them [4]. A similar device to the out-of-plane converter was proposed by Sterken *et al.* [45]. The device consists of two capacitors, one stationary, and one mobile. As the capacitance of the system varies via the free capacitor, the change in capacitance will cause a current in a similar manner to the previously discussed designs. This device is capable of generating 100 μW while excited at 1,200 Hz [45]. The design of this micro generator was optimized to allow the operational frequency to be as close as possible to the natural frequency of the generator. Therefore, the generator was able to be operated near resonance, maximizing the displacement of the free capacitor, thereby maximizing the generation possible. As with previous incarnations of electrostatic MEMS-based generators, this design requires a polarization source to charge the capacitors prior to generation.

A comb-based electrostatic microgenerator was developed by Ma *et al.* [46] using an out of plane, or vertical, comb drive rather than an in plane, or horizontal one. In this case, a variable capacitor is formed from an insulated floating heavily doped poly-silicon electrode and a metal electrode of similar geometry suspended at a specific gap, directly over top of the floating poly-silicon electrode. The gap between electrodes does not change. The microgenerator is actuated horizontally, causing the required capacitance change in order to produce electricity. The capacitance change is largely caused by the fringing of dielectric fields, rather than the more direct overlapping of previous designs. This microgenerator was capable of producing 65 nW of power under a resistance load of 50 MΩ, driven at near-resonance, at a displacement of 2.2 μm [46]. This specific generation scheme uses capacitor polarization that is provided by electron tunneling, similar to the process found in non-volatile memory devices [47].

### 2.5. Electromagnetic Conversion

**Figure 5.**
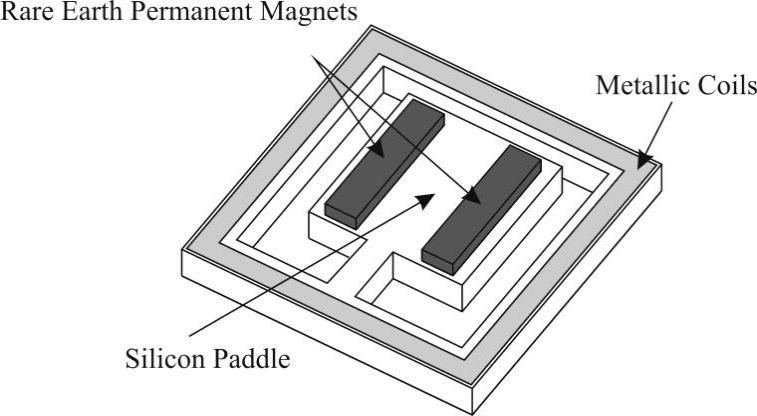
Schematic of a Sample Electromagnetic Generator.

Electromagnetic generation has been used to generate power since the discovery of electromagnetic induction by Faraday, which led to the development of the first magneto by Pixii [48]. Since that initial discovery, the principle of generating power from oscillating magnetic fields and a conductor has been extensively used both in large and small scales. Electromagnetic vibration-to-electricity conversion is a fundamentally regenerative power generation scheme—as long as the actuation is ambient. Electromagnetic generation has even bridged the gap into MEMS as shown by Roberts *et al.* [2] which are using a MEMS-based electromagnetic generation scheme to augment the power supply for pacemaker batteries in clinical trials. The electromagnetic MEMS-based generator is schematically shown in Figure 5.

Generally, electromagnetic microgenerators consist of an arrangement of permanent magnets and metallic coils that move relative to one another. As schematically seen in the Figure 5, the device consists of an arrangement of magnets placed on a vibrating beam. A coil is contained within the silicon beam layer, running the perimeter of the etched well. As the beam vibrates out of plane, the magnetic field oscillates relative to the coils on the well’s edge, causing the coils to be subject to a magnetic flux. The flux imparts an electromotive force on the coils, causing a current to flow in the coils. This device is capable of significant power generation at operating frequencies of 30–350 Hz, well below the reported natural frequencies of the device, which range from 6.4 to 12.6 kHz [49]. Since the performance of an electromagnetic microgenerator is tied to the magnetic flux that is produced from vibration, optimizing the amount of vibration that the microgenerator receives is important. Optimizing the vibration characteristics of the electromagnetic microgenerator, such as improving the linear behavior, reducing the parasitic damping, and tuning the frequency response of the generator to the ambient vibrations that the microgenerator is subject to is of high importance [50]. To this end, both the geometry and the materials that are used in the microgenerator must be optimized. Silicon based materials, such as Si, SiO_2_, and Si_3_N_4_ are preferable to polymeric materials, such as Kapton due to lower mechanical losses and lack of spring stiffening effects at large excitation amplitudes [51].

Reissman *et al.* [52], have developed a similar method of generating electricity through electromagnetic induction on a MEMS scale. As in previous devices, an oscillating magnetic field is used to induct electrical current, through the electromotive force, into a MEMS-scale coil. A NdFeB permanent rare earth magnet provides the strong magnetic fields required for this microgenerator. The permanent magnet is suspended via a rigid beam 2 mm above a micro-coil of copper, fabricated from CMOS processes. In this configuration, the fringing of the suspended NdFeB magnet is supplying the magnetic flux to the copper micro-coil. At a frequency of 27 Hz, the device is able to produce 12.5 μW of power per copper coil layer [52].

Serre *et al.* [50] developed a membrane based microgenerator that uses Kapton, a polymer-based membrane. The Kapton membrane is a suitable material for low frequency actuation applications, having a Young’s Modulus much lower than other possible membrane materials such as silicon. A Kapton membrane of 127 μm thickness was used to suspend a NdFeB rare earth magnet inside a micromachined well. Coils were deposited on the top surface of the wafer, above the wells. A prototype microgenerator, with a 7 × 7 × 4 mm^3^ magnet and a 13 × 13 mm^2^ Kapton membrane with a resonant frequency of 360 Hz was able to produce a peak power of 45 nW [50]. An optimization of this generator was undertaken in order to increase the power output [51]. The geometry of the Kapton membrane was optimized to provide greater displacement to the permanent magnet, in order to maximize the magnetic flux that would be produced. Unfortunately, parasitic damping, caused by spring stiffening effects increases as the amplitude of the membrane displacement increases, adding losses to the system with increased displacement. To further increase the power generation that this type of microgenerator can produce, thicker electroplated copper coils have been suggested by Serre *et al.* to increase the peak power generation from 45 nW to between 60 to 120 μW [51].

A rotary electromagnetic generator was produced by Pan *et al.* [53]. The microgenerator consists of two disks, one disc consisting of an 8-pole NdFeB magnet, and the other consisting of various layers of copper multipolar coils with a line width of 30 μm. These two discs were separated by one millimeter - the magnetic disc suspended on a rotary mechanism, while the coils attached to a static platform. In this case, four layers of copper coils were used to increase the generation potential of the rotary electromagnetic generator. Running the rotating platform at 150 Hz, the maximum induced voltage from a four layer coil disc is 111.2 mV, with a maximum power output of 386.42 μW. Another rotary generator was developed by Herrault *et al.* [54] that uses an air turbine as an actuation mechanism for its rotary microgenerator. As with other electromagnetic microgenerators, a NdFeB permanent magnet will be used to provide the strong magnetic field required. The design of the microgenerator in this case is similar to Pan *et al.* [53], however the stator coils are of a more complex design. Coils that will experience the same electrical phase are connected, thereby increasing the electricity generated at a specific electrical phase to be maximized. The poles of the coil assembly were equally spaced, depending upon the number of coils that were used in the stator design. In addition, to maximize the generated electric power with small diameter rotary microgenerators the speed at which the rotor will rotate increases, in comparison to macro scale devices. This device is driven at 392 kRPM, producing 6.6 mW of electrical power. These microgenerators produce a fair amount of electricity; however rotation is not a convenient motion of vibration to harvest energy from. To provide the necessary mechanical rotation for most electromagnetic generation schemes a MEMS-based turbine or rotational engine will be required.

### 2.6. Piezoelectric Conversion

**Figure 6.**
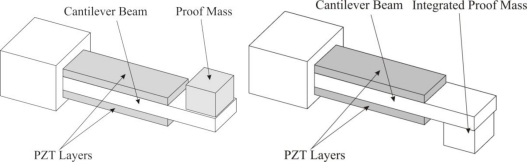
Schematic of a Laminated Piezoelectric Beam Micro Generator [55].

Piezoelectric generation is a well researched method of harvesting power from mechanical vibrations. When the crystal structure of the piezoelectric material is loaded, the micro-structure of the crystal is distorted. In order to maintain electrical equilibrium within the crystal the electrons become mobile and shift, creating a current. This is referred to as the direct piezoelectric effect. Alternatively, the exact opposite phenomenon, the converse piezoelectric effect, can also take place. For micro-generation, the direct piezoelectric effect is used to convert vibration to electricity. The direct piezoelectric effect is used for microgeneration and sensing purposed, while the converse piezoelectric effect is used mainly for actuation. Piezoelectric generation is frequency dependant, maximized as the frequency at which the system is driven is at resonance [55], where the displacement is maximized. Cantilever beams are the most convenient arrangement of piezoelectric material for generating purposes because it allows for the 31-mode of the piezoelectric material to be accessed easily, maximizing the voltage output of the piezoelectric material, especially in low strain realms [56], as shown below in Figure 6 [55].

**Figure 7.**
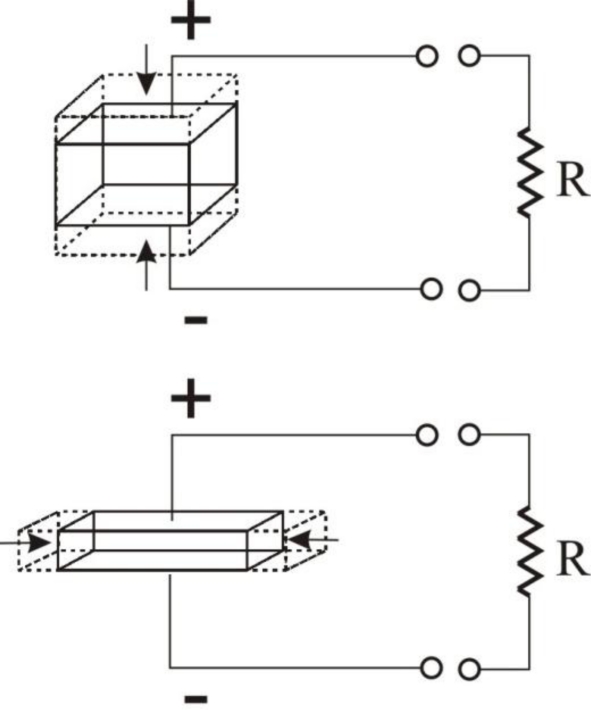
The 33-mode (top) and 31-mode (bottom) Modes of a Piezoelectric Material [55].

Piezoelectric materials have multiple modes of operation, as shown below in Figure 7. As seen in this Figure, the modes of a piezoelectric material simply refer to direction of mechanical force applied and electric charge collected. The top of Figure 7 shows the 33-mode of a piezoelectric material, where the charge is being collected on the surface perpendicular to the polarization axis while the mechanical force is applied along the polarization axis. The bottom of Figure 7 shows the 31-mode of a piezoelectric material, where the charge is being collected on the surface perpendicular to the polarization axis, and the mechanical force is being perpendicular to the polarization axis [57]. These arrangements can be used in order to maximize generation depending upon the loads placed on the piezoelectric material.

The piezoelectric microgenerator requires a piezoelectric film to convert the displacement and strain into electricity through the piezoelectric effect. There are three materials that can be deposited as thin films for this application, lead zirconate titanate (PZT), zinc oxide, and aluminum nitride. In literature, PZT is the dominantly used for power generation purposes. ZnO and AlN are more commonly used in actuation and sensing. In terms of microfabrication, ZnO and AlN are less complicated and have fewer equipment contamination issues than PZT. The material properties of these thin films are shown below:

As can be seen from Table 1, the piezoelectric coefficients for a variety of PZT materials are much higher in magnitude than AlN and ZnO thin films. For power generation applications higher piezoelectric coefficients, especially the d_31_ coefficient, are desirable [58]. However, biocompatibility of the AlN and the ZnO based microgenerators can be desirable for implantable sensing applications.

The majority of the research into piezoelectric microgenerators centers on optimizing the performance and efficiency of the generator. Specifically, the work deals with optimizing the conditioning circuits that are used to collect and store the generated power and optimizing the amount of power generated by adding mass to the system [55,56,59]. The power that a piezoelectric generator is capable of generating is directly proportional to the strain the piezoelectric crystals are subject to, as shown by the equations below:

(5) $$\sigma =Eɛ=Eu_{x}$$

(6) $$\left[T\right]=\left[c\right]\left[S\right]-\left[e^{t}\right]\bar{E}$$

where, in (6) *[T]* is the Stress Field Tensor, *[c]* is the Elastic Stiffness Tensor, *[S]* is the Strain Field Tensor, *[e^t^]* is the transpose of the crystal symmetry tensor specific to the piezoelectric material, and *E* is the Electric Field Vector. To maximize the strain, the displacement that the generator undergoes must be maximized as well. In addition, piezoelectric generation is maximized as the frequency at which the system is driven is at resonance [55].

Roundy *et al.* [60] have examined the properties and generation potential of the piezoelectric cantilever microgenerators. The generators developed by Roundy *et al.* are not considered to be MEMS devices, but important results have been gained from their work. Their microgenerators were limited to 1 cm^3^ total volume, using tungsten proof masses to tune the frequency-based characteristics of the microgenerators. Roundy *et al.* observed several key results to aid in the optimization of this type of MEMS-based generator. First, the microgenerator’s resonant frequency should be as close to the operational frequency of the generator as possible in order to maximize power output [60]. This ensures that the cantilever-based microgenerator will experience maximum displacement and strain, thereby maximizing the power generated. Additionally, the power output of the system is inversely proportional to the driving and resonant frequency of the device [60]. Moreover, the power output of the microgenerator is proportional to the seismic mass of the system. Higher mass in the system helps reduce the natural frequency of the microgenerator. Furthermore, Roundy *et al.* also determined that the energy removed from the generator will act as mechanical damping to the system, due to the piezoelectric coupling in the system [60]. The opposite is also possible; increasing the electrically induced damping to the system will maximize the power output. Roundy *et al.* were able to produce cantilever-based piezoelectric microgenerators that were able to produce 375 μW from driving vibrations of 2.5 m/s^2^ at 120 Hz [60].

Aluminum nitride based cantilever systems are also being explored for piezoelectric microgeneration applications. As discussed by Elfrink *et al.* [61], the major advantage of using AlN-based piezoelectric microgeneration scheme, in comparison to a PZT-based one, is the higher optimum load resistance of the AlN in comparison to the PZT. With an optimum load resistance, the generator will produce the optimum power. For AlN, Elfrink reported an optimum load resistance of 0.1–1.0 MΩ, where PZT based microgenerators generally have optimum load resistances of a few kΩ. This difference in load resistance causes AlN to generally have higher output voltages that PZT (for equivalent power output), which may be desirable for certain power generation applications. The AlN-based cantilever generators are fairly large for MEMS-based generation, up to 7 × 7 mm in footprint, with beam thicknesses of approximately 45 μm, which allowed for natural beam frequencies as low as 277 Hz. The maximum power output from this scheme was 60 μW at an operational/natural frequency of 572 Hz. To be efficient, this microgenerator needs to be packaged in a vacuum, since air damping in the required encapsulation scheme causes significant damping and generation losses.

In addition to the cantilever type piezoelectric microgenerator, membrane-based generators are being investigated for both implantable and ambient uses. Generally, a circular membrane of lead zirconate titanate (PZT) is used [62] due to its axisymmetry. In biomedical applications, a circular membrane piezoelectric microgenerator can be tuned to actuate from pressure differences found in the body, such as those generated by breathing, muscle contractions or blood flow. A circular piezoelectric microgenerator [62] was designed to be actuated from the pressure difference (40 mmHg) that is produced from a typical human pulse. This device was able to generate 61 μW experimentally from the 40 mmHg pressure load. Ramsay and Clark [56] have also examined using blood pressure as a power source for piezoelectric membrane microgenerators. It has been reported that the power available from variations in blood pressure is as high as 0.373 W [63]. Even with the relatively low conversion efficiency of 34% of the PZT-5A material used in the analysis, it is theoretically possible to produce membrane-based piezoelectric microgenerators that could easily provide 10 mW of continuous power. However, Ramsay and Clark discovered that although the generation potential was there, the size of the membrane would be a limiting factor in generation. With membranes limited to 1 cm^2^ it was not possible to produce 10 mW of continuous power. Using blood pressure (40 mmHg) alone as an actuation method for the piezoelectric microgenerator, and limiting the size of the membrane in the microgenerator to 1 cm^2^, it was possible to continuously supply microwatts of continuous power, while being able to provide milliwatts range power intermittently when the displacement of the microgenerator is maximum.

In addition to piezoelectric membranes for biomedical applications, a PZT microfiber generator has been developed by Ishisaka *et al.* [64] in which the contractions of a heart muscle are used to actuate the piezoelectric microfiber generator. The piezoelectric microfibers are fabricated by depositing PZT onto a platinum wire, and then plating the wire with nickel in order to complete the electrical circuit. A PDMS membrane is then placed between the fiber and the heart muscle to provide biocompatibility. As the heart muscle contracts, the PDMS membrane is deflected, which in turn causes the embedded PZT microfiber to deflect as well. In experiments, cultured cardiomyocytes were used for actuation. These lab-grown cells actuated the piezoelectric generator at a frequency of 1.1 Hz, producing between 40–80 mV, for a single ∼100 μm fiber. The strength of this microgenerator is the high biocompatibility of the PDMS membrane encapsulation that prevents contact between the PZT and the cardiomyocytes. Not only is the material highly flexible, allowing for actuation, it is completely biocompatible. Arrays of PZT microfibers may be used to increase generation in this application.

Another fiber-based piezoelectric vibration scheme uses Zinc Oxide nanowires to generate electricity on a micro scale. The ZnO nanowires are grown using a wet chemistry method to deposit the nanowires on a plastic substrate [65]. The wet chemistry method can be altered for different orientations and densities of ZnO nanowires. The flexibility of the plastic substrate allows for the ZnO nanowires to be used in a flexible application, such as implantable biosensors [65]. The flexibility will allow for the substrate to flex with muscles and tissues, since in this case, this is generally an area-based method of generation. ZnO wires with a 300 nm diameter, 1 μm long, can produce an output power of approximately 5 pW at 45mV, under a 5 nN contact force load through AFM actuation [65]. Xu *et al.* [66] have developed ZnO nanowire-based array microgenerators deposited on Kapton, using a similar process as above. The deposited ZnO nanowire array is then encapsulated by a soft polymer, such as photoresist in order to protect the nanowires from the environment. This generator was capable of generating 1.26 V at a strain of 0.19%, potentially being capable of charging a AA battery [66], producing a peak power output of 2.7 mW/cm^3^. A similar microgenerator has been used in clinical trials by Li *et al.* [67] to generate power using a ZnO nanowire generator in an *in vivo* application. This microgenerator has bigger nanowires than the previous two microgenerators having a diameter of 100–800 nm and length of 100–500 μm [67], packaged in a similar manner using flexible polymer materials as above. The microgenerator was implanted on the diaphragm muscle and heart of live rats in order to test the potential of this type of microgenerator in an implantable application. For the expansion and contraction of the diaphragm during breathing, the ZnO nanowire generator was able to generate 1 mV at 1 pA [67]. The generator was able to generate more power from the heart beat of the rat, generating 3 mV and 30 pA [67]. Although this experiment produced significantly less power than the previous studies that used purely mechanical stimulation, it is an important first step for ZnO nanowire-based *in vivo* microgenerators.

## 3. Discussion

For implantable biosensing power systems applications, there are a variety of possible generation techniques that could allow existing power systems to be supplemented or replaced, allowing for long term and autonomous operation. Energy scavenging techniques are generally more suitable for this type of operation, since no additional fuels or stimuli would be needed for continuous power generation. In addition, with suitable use of sleep modes, power conditioning circuits, and power storage through thin film batteries or capacitors, an entirely self contained power system could be easily integrated into wireless implantable biosensing platforms. Not only would this allow for increased operational time, additional components could be added to the implantable system, allowing for increased functionality, without adversely affecting the total lifespan of the implant.

In addition to being regenerative, the MEMS-based microgenerators must also be biocompatible for implantation. Either all components must be suitable for implantation, or sufficient packaging must be in place to prevent possible biocompatibility issues. This is usually accomplished by packaging the microgenerators or biosensing platform in polymeric or silicone gel encapsulation [68]. This type of packaging is required to ensure biocompatibility both for the patient and the implant. The patient must be protected from possible cytotoxic materials, while the implant itself must be protected from environmental factors *in vivo* that may reduce their generation effectiveness. The majority of materials used in MEMS devices, including silicon, silicon oxide, polysilicon, silicon nitride, titanium, and some photoresists, such as SU-8, have been proven to be non-cytotoxic [68]. However, there are some materials, such as lead zirconate titanate (PZT) that can be cytotoxic if improperly packaged. In addition, the topology of various MEMS devices may cause many sharp edges to be present on the cellular level, allowing for the potential of localized cell damage through direct contact. For these reasons, MEMS devices used in implantable biosensing are usually packaged in a biocompatible manner, regardless of the cytotoxicity of the materials involved.

The type of MEMS-based generator chosen for a specific implantable application depends solely on the type of input energy that is available in the specific implantation site. Ambient light, the most plentiful source of input energy available, suffers from major issues with light intensity. For example, the efficiency of a photoelectric generator suffers greatly from diminished light intensity, up to a 94% loss in efficiency from not being in direct sunlight [4]. For implantable systems, this could be troublesome, since light does not penetrate very deeply subcutaneously; therefore the efficiency of the photoelectric based systems would be additionally diminished for implantable systems. Ambient heat generated by the body is also an abundant energy source for implantable systems. The body constantly generates heat from the various biological processes required to sustain life. To maximize efficiencies, the areas of the body with maximum temperature differentials should be prioritized, for example, where blood vessels are in close proximity to the exterior of the body such as the neck, wrists, and ankles. Ambient vibration in the body can be any movement, voluntary or involuntary within the body. This can range from the displacements from a beating heart, expanding diaphragm, a pulse from an artery, or shock/movement from walking. Each of these vibrations can be harvested by a variety of generation schemes as long as careful design allows for the capture of these motions. This may involve specific tuning of the microgenerator to a specific actuation frequency range through design or active stiffening control. In addition, arrays of vibration driven microgenerators may be arranged to capture a wide frequency band of actuation, each microgenerator in the array tuned to a specific subsection of that frequency band. Table 2 briefly states the advantages, disadvantages, and power generation potential of each type of power generation explored. As shown in the Table, photovoltaic schemes are perhaps the least suitable for implantable biosensing applications. The requirement for direct light for optimum generation makes the photovoltaic class of microgenerators somewhat impractical for implantable biosensing applications.

Unless high intensity light is transported subcutaneously to an implanted photovoltaic system via fiber optics or the power generated on the surface of the skin can be transmitted to the implant, as discussed in Section 2.1, photovoltaic generation will suffer from low conversion efficiency and power output. However, if the generator could be worn or integrated into a piece of clothing and the generated power transmitted into the body, photovoltaic generation may be suitable as a generation technique for implantable biosensing. Although there have been advances in the technology to allow for improved conversion rates and adaption of the technology to overcome the challenges inherent to the generation scheme as discussed in Section 2.1; the majority of implantable biosensing applications are not applicable for use with photovoltaic schemes.

Thermal-to-electricity generation is an interesting alternative to batteries for biosensing applications due to of the availability of ambient heat energy and the reasonable power generation potential at optimal generation sites. Direct thermoelectric generation is an energy scavenging technique that can be effective with small temperature differentials, while maximized with high temperature differentials. With increasing efficiency of various thermopile designs, as discussed in Section 2.2, the average size of direct thermoelectric generators have decreased, making them increasingly attractive for implantable biosensing applications. Indirect thermal to electricity conversion may be attractive for implantable biosensing as long as the heat required to produce the mechanical displacement by these systems could be gained from methods other than combustion. Combustion, *in vivo*, could have significant packaging challenges such as reactant and product handling challenges, not to mention the localized heating. Keeping this in mind, the methods discussed in Section 2.2 using combustion as a heat source may not be directly applicable to implantable biosensing applications. In addition, the implantation location of the thermoelectric-based microgenerators is of great importance, since the body has an inhomogeneous thermal profile. The possible applications of thermoelectric generation may be limited by the thermal profile of the body at the required implantation site. Locations within reasonable proximity of major blood vessels may be the most suitable for implantable biosensing, since blood itself plays a major role in thermal regulation within the body. Without a sufficient thermal gradient at the implantation site, it may not be feasible to use thermoelectric generation to produce sufficient operational power in implantable biosensing applications.

Micro fuel cells are a suitable power generation scheme for implantable biosensing as long as the fuel cell is regenerative and biocompatible. Regenerative fuel cells, such as glucose based fuel cells, can be implanted for long term operation since the fuel and oxidation reagent is replenished constantly through the electrochemical reactions that generate power. In addition, glucose is readily available in the body, thus additional fuel for glucose-based micro fuel cells is available if needed. As discussed in Section 2.3, micro fuel cells using non-biocompatible reactants generally produce more power than those using biocompatible fuels. There are intrinsic difficulties when dealing with non-biocompatible reactants and implantation, such as the possibility of insufficient packaging causing the potential leaching of reactants out of the fuel cell damaging surrounding tissues. However, the advances in miniaturization, components, composition, and packaging discussed in Section 2.3 may eventually lead to a higher output biocompatible fuel cell utilizing non-biocompatible fuels. The smaller and more efficient a micro fuel cell, the more applicable it will be to implantable applications. Micro fuel cells are the only location independent MEMS-based generation technique, not relying on a specific implantation location or physical phenomenon to supply the specific input energy required to produce power. As long as the implantation location can accommodate the size of the micro fuel cell and does not impart any specific loading to the micro fuel cell that may damage its packaging, it would be applicable for implantable biosensing techniques.

Electrostatic MEMS-based generation requires very specific physical motions in order to produce an optimum amount of electricity. Implantation locations that undergo predictable planar displacements or vibrations are ideal for electrostatic generation. A specific method of electrostatic generation, as discussed in detail in Section 2.4, can be chosen in order to maximize the generation attained from a specific known planar actuation. The strength of electrostatic MEMS-based generation lies within the multiple actuation directions and arrangements that are possible. This is important for biosensing applications, since conceivably, electrostatic generation could be used in a multitude of orientations, many of which would not be possible with other vibration-based generation schemes. However, rotation and off axis motion is troublesome for electrostatic based schemes, causing potential damage through collision and stiction. In addition, in order to generate power, the electrostatic generator must be pre-charged in order to take full advantage of the motion-induced capacitance changes. Efficiency and generation of these systems are improved by using self-charging mechanisms such as electrets and charge pumps, as discussed in Section 2.4. Using these self-charging mechanisms, the electrostatic MEMS-based microgenerators are a self-contained system suitable for a multitude of implantable biosensing applications where known planar motions and vibrations are present.

Electromagnetic generation is an energy scavenging technique that is currently used in some high profile implantable applications, such as generating supplementary power for pacemaker batteries [2]. As with electrostatic-based generation, electromagnetic generation has a directional actuation dependence, therefore knowledge of the implantation site conditions is critical. However, electromagnetic generation is significantly more robust than electrostatic, since any out of plane actuation is usable—off axis actuation is not detrimental or damaging to the electromagnetic generator. As discussed in Section 2.5, optimization of the power generated through this strategy is achieved by maximizing the magnetic flux experienced by the coils of the generator. Rotary electromagnetic generation is a well known method to maximize the magnetic flux possible on a macro-scale. Although a novel MEMS application and relatively high power density scheme, rotary motion may be difficult to supply to the microgenerator in comparison to linear actuation. Rotary MEMS engines may be used to accomplish this, although their applicability *in vivo* would be significantly limited. Therefore, linearly actuated electromagnetic MEMS-based microgeneration techniques would have more possible implantable applications than rotary ones. Electromagnetic generation, although having reasonable power densities, can have scaling issues with miniaturization. As the scale of the electromagnetic generators decreases, it has been suggested by Beeby *et al.* [70] that the power generation potential of the microgenerator decreases as well. The power density and power generation of electromagnetic based schemes can lag behind other similar techniques in this size regime.

Piezoelectric based generation is suitable for implantable biosensing having high power densities allowing for sufficient power generation for many applications. For high power density piezoelectric MEMS-based generation using PZT films, the major challenge in regards to implantable applications is biocompatibility. As discussed in Section 2.6, advancements toward biocompatible PZT-based piezoelectric generators have been made. Biocompatible packaging, such as PDMS will be required in order to allow piezoelectric schemes to be used in implantable biosensing applications. Although AlN and ZnO-based microgenerators have less power generation potential according to the material properties of the respective piezoelectric films, important advances have been made towards using microgenerators based on these materials in *in-vivo* applications. The recent advances and inherent biocompatibility of these materials is making AlN and ZnO-based piezoelectric generators an interesting alternative to PZT-based systems for implantable applications. As with electromagnetic generation, off-axis actuation is not potentially dangerous to the operation of the microgenerator. It is non-optimal, but will not prevent generation or cause damage. As discussed in Section 2.6, optimization of the frequency response is required in order to maximize the power generated. The vibrations that actuate the piezoelectric microgenerators can be low frequency and low amplitude, which are easily achievable for implantable biosensing. The piezoelectric microgenerators can be tuned to specific frequency responses found at the implantation site, allowing the microgenerators to be designed to maximize the local power generation possible at an implantation site. In addition, being able to take advantage of pressure variations and displacements, such as an arterial pulse or a flexing muscle is attractive for biosensing applications.

## 4. Conclusions

There are many alternatives available to augment or replace existing conventional battery-based systems for powering implantable biosensors. These systems include photovoltaic, thermovoltaic, micro fuel cells, electrostatic, electromagnetic, and piezoelectric based microgenerators. For implantable biosensing applications the most suitable microgenerators are based on energy scavenging, using ambient energy sources such as heat and vibration to produce electricity. In addition, the microgenerator must be able to easily operate as a surgical implant and must be completely biocompatible. Therefore, micro-generation schemes based upon photovoltaic microgenerators and hydrocarbon-based micro fuel cells may not be suitable for microgeneration in implantable biosensing applications.

Determining the correct microgeneration scheme for a specific implantable biosensing application can be a difficult task. Power density is important for implantable biosensing power generation. The smaller a microgenerator can be in this case, the less invasive the implant. Therefore, microgenerators with high power densities, for example, those using piezoelectric and electromagnetic schemes would be the least invasive for a vibration-based application, requiring the smallest volume to provide a necessary power level. In situations where more than one generation physics can be utilized, power density should be a major consideration in order to reduce the power system’s overall size.

The most significant factor used in determining the most applicable microgeneration technique to use in a specific implantable biosensing application is ambient energy available at the implantation site. Whether the energy available is solar energy, heat, or vibration, the microgeneration technologies studied are exclusive in terms of their input energy. If the implantation site is near the surface of the skin, it may be advantageous to consider photovoltaic schemes. If high thermal gradients are present at an implantation site, thermovoltaic schemes will outperform the other available technologies. In areas of high motion and vibration, the vibration-based energy harvesting techniques, such as electrostatic, electromagnetic, and piezoelectric schemes will be the optimum choice. For example, if no harvestable energy is present at a specific implantation location, it will be more advantageous to focus on micro fuel cell technologies than other alternatives. A well developed understanding of the specific input energies available at an implantation site will lead to the best possible choice of a generation technology for the specific *in vivo* application.

## Figures and Tables

**Table 1.** Thin Film Piezoelectric Materials.

**Thin Film Piezoelectric Material**	**Fabrication Method**	**Fabrication Difficulty (Easy/Medium/Difficult)**	**Piezoelectric Coefficient d_31_ (pC/N)**	**Piezoelectric Coefficient d_33_ (pC/N)**
Aluminum Nitride (AlN)	Sputtering	Easy (Sputtering)	0.7	2.0
Lead Zirconate Titatnate (PZT)	Sputtering, Sol-Gel Deposition, Metapl Oxide Chemical Vapor Deposition (MOCVD)	Easy (Sputtering) Medium (Sol-Gel Deposition, MOCVD)	−60 (PZT-2) −171 (PZT-5) −220 (PZT-5J)	152 (PZT-2) 374 (PZT-5) 500 (PZT-5J)
Zinc Oxide (ZnO)	Sputtering	Easy (Sputtering)	−5.43	11.67

**Table 2.** Comparison of Power Generation Techniques for Implantable Biosensing Applications.

**Method of Micro-generation**	**Advantages**	**Disadvantages**	**Power Generation Potential**	**Input Energy Source**	**Applicability to implantable applications**
Photovoltaic	Regenerative, abundant power source.	Efficiency and output is tied to light intensity.	500 μW [11]–1 W [12]	Light/Photons	Applicable where sufficient light intensities are present. Not Applicable otherwise.
Thermovoltaic	Regenerative	Size Requires large temperature difference for efficient generation.	4.5 μW–100 μW [16] (Thermopiles) 0.8 μW [22] (P^3^ Micro-heat engine)	Ambient or supplied heat.	Applicable
Micro Fuel Cells	Can be regenerative. Reasonable energy density.	Hydrocarbon fuels (highest energy) are not biocompatible.	50 μW/cm^2^–430 μW/cm^2^ [25] (Glucose based) 9 mW/cm^2^–750 mW/cm^2^ [29,35] (Hydrocarbon Based)	Supplied fuels such as Glucose or Hydrocarbons	Glucose based micro fuel cells are applicable. Hydrocarbon micro fuel cells are not.
Electrostatic	Can be regenerative with electrets and charge pumps.	Requires energy to produce energy.	20 μW/cm^2^–116 μW/cm^2^ [4] (In-plane gap closing type) 100 μW/cm^2^ [45] (out-of-plane type)	Ambient or supplied vibration.	Applicable
Electromagnetic	Regenerative High power Density.	Poor length-scale based scaling.	12.5 μW [52] (Cantilever) 45 nW [50] (Membrane) 386.46 μW [53]–6.6 mW [54]	Ambient or supplied vibration.	Applicable
Piezoelectric	Regenerative High power density. Customizable	Possible bio-compatibility issue. Highly frequency dependant.	375 μW [60] (Bimorph) 10 mW [69] (Membrane) 2.7 mW/cm^3^ [66] (ZnO Nanowire)	Ambient or supplied vibration.	Applicable
